# Histone Acetylation Changes in Plant Response to Drought Stress

**DOI:** 10.3390/genes12091409

**Published:** 2021-09-13

**Authors:** Shuang Li, Xu He, Yuan Gao, Chenguang Zhou, Vincent L. Chiang, Wei Li

**Affiliations:** 1 State Key Laboratory of Tree Genetics and Breeding, Northeast Forestry University, Harbin 150040, China; hxnefu@163.com (X.H.); yuangao691@gmail.com (Y.G.); zhouchenguang@nefu.edu.cn (C.Z.); vchiang@ncsu.edu (V.L.C.); weili2015@nefu.edu.cn (W.L.); 2 Forest Biotechnology Group, Department of Forestry and Environmental Resources, North Carolina State University, Raleigh, NC 27695, USA

**Keywords:** drought stress, histone acetylation, histone acetyltransferases (HATs), histone deacetylases (HDACs)

## Abstract

Drought stress causes recurrent damage to a healthy ecosystem because it has major adverse effects on the growth and productivity of plants. However, plants have developed drought avoidance and resilience for survival through many strategies, such as increasing water absorption and conduction, reducing water loss and conversing growth stages. Understanding how plants respond and regulate drought stress would be important for creating and breeding better plants to help maintain a sound ecosystem. Epigenetic marks are a group of regulators affecting drought response and resilience in plants through modification of chromatin structure to control the transcription of pertinent genes. Histone acetylation is an ubiquitous epigenetic mark. The level of histone acetylation, which is regulated by histone acetyltransferases (HATs) and histone deacetylases (HDACs), determines whether the chromatin is open or closed, thereby controlling access of DNA-binding proteins for transcriptional activation. In this review, we summarize histone acetylation changes in plant response to drought stress, and review the functions of HATs and HDACs in drought response and resistance.

## 1. Drought Response Physiology and Strategies

Drought is a major concern for agriculture and forestry productivity, and it is predicted to increase in duration and severity due to the global climate change [1]. Drought is often accompanied by productivity loss; long-term severe drought stress may even lead to plant mortality [2,3]. Because of the sessile nature, plants have evolved sophisticated strategies to develop drought resistance for survival [4,5,6,7,8,9]. Increasing water absorption and conduction, reducing water loss, conversing growth stages are effective ways for plants to survive under drought conditions [3,4].

Roots are key tissues of plants that absorb and uptake water from the soil to aboveground organs. Under drought conditions, plants develop a better root system by adjusting the depth and density of their roots to increase water absorption from the soil [4,6,7]. For example, because deep roots are effective at enhancing water capture and increasing the water uptake range under drought stress, plants tend to develop more lateral roots in the wet soil side than the dry soil side [4,6,7].

The stem of plants is an important aboveground tissue for water conduction. Plants also change cell morphology of the stem to enhance water conduction [4]. The characteristics of vascular cells in the stem of plants are the most important factors to resist embolism and maintain water conduction [10,11]. Small changes in size, quantity, distribution, spacer and other characteristics of the vascular cells can greatly affect the water conduction efficiency [12]. For example, a larger vascular cell aperture increases the efficiency of water conduction, but also increases the production of cavitation [13]. Under drought stress, plants balance water conduction efficiency and cavitation risks to create optimal characteristics of vascular cells for drought resistance [2,13].

Plant leaves are the main tissues of water loss [4]. The morphological and physiological characteristics of leaves are crucial to reducing water loss. Upright and rolling leaves are effective mechanisms of drought resistance for receiving less radiation and therefore reducing water loss [4]. Leaves with traits such as thicker wax layer, smaller and denser stomata are always a benefit to support water conservation. Stomata are the vital organs for exchanging gas and water between the plant and the external environment. Under drought conditions, plants often reduce water loss through rapid stomatal closure [4].

In addition, the conversion between vegetative growth and reproductive growth is also an important drought resistance strategy [4]. For example, it is a good strategy for plants to complete their life cycles before severe drought stress, so early flowering may take place to prevent the plants from abortion [3,4].

## 2. Molecular Response to Drought: The ABA Drought Response Pathways

The drought response in plants is frequently initiated by signal pathways, such as abscisic acid (ABA), acetic acid, jasmonic acid (JA) pathways, etc. [14,15,16,17,18,19]. ABA biosynthesis and signaling pathway is the most important biochemical and molecular triggering response and resistance to drought in plants. When plants sense water deficiency in soil, ABA is synthesized in roots and released into aboveground organs to trigger plant drought response [18]. There are two pathways, ABA-dependent and ABA-independent, that are associated with the regulation of drought-responsive genes [15,16]. In the ABA-independent pathway, dehydration-responsive element binding protein 2A (DREB2A) is a key transcription factor which plays pivotal roles in drought response [20,21]. In this review, we focus on the ABA-dependent signaling pathway.

**Figure 1:**
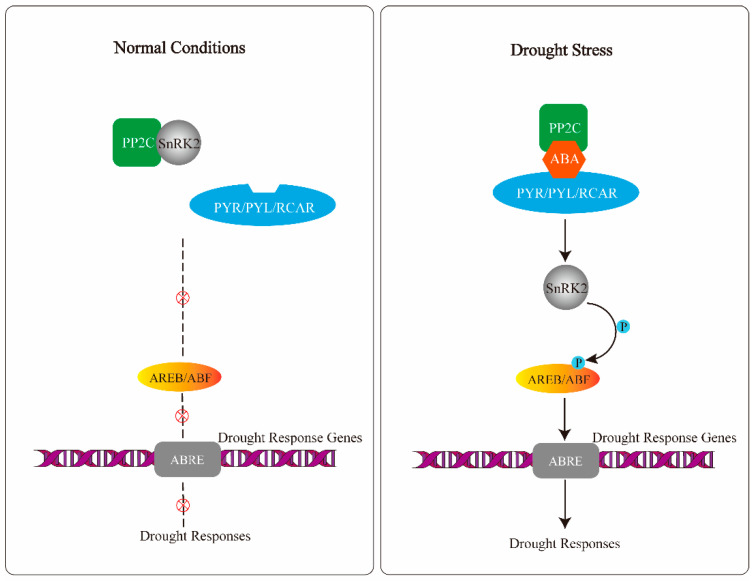
ABA-mediated signal pathway in plants for drought responses.

In the ABA signal pathway, pyrabactin resistance/pyrabactin resistance-like/regulatory component of ABA receptors (PYR/PYL/RCARs), SNF1 (sucrose non-fermenting 1)-related protein kinase 2 (SnRK2) and protein phosphatase 2C (PP2C) are the three core components. PYR/PYL/RCARs are ABA receptors that have the ability to recognize and bind ABA directly. SnRK2 is a positive regulator of downstream proteins, such as transcription factors, through phosphorylation of the protein. Under normal conditions, ABA is absent, and PP2C associates with SnRK2 to prevent SnRK2-mediated phosphorylation of downstream drought response genes, thereby deterring the initiation of drought responses [14,15]. Under drought conditions, ABA is accumulated rapidly, PYR/PYL/RCAR receptors recognize and bind to ABA and interact with PP2C. This process leads to the release and activation of SnRK2 [14,15]. The activated SnRK2 phosphorylates downstream drought response genes, such as abscisic acid-responsive element binding protein/ABRE binding factor (AREB/ABF), are the best characterized drought-responsive transcription factors [14,22]. AREB/ABF bind to ABRE (ABA-responsive element) containing ABA-responsive genes and activate their expression to initiate drought responses (Figure 1) [13,14,23].

It is well described that drought response in plants is associated with the regulation of drought response genes, such as ABA-responsive genes [16,24]. These processes rely on complex transcriptional regulation. An increasing amount of evidence indicates that epigenetic modifications also play significant roles in drought response regulation [25,26,27,28,29]. However, there has long been a missing link between transcriptional and epigenetic regulation mechanisms.

## 3. Regulation of Histone Acetylation in Drought Response

**Figure 2:**
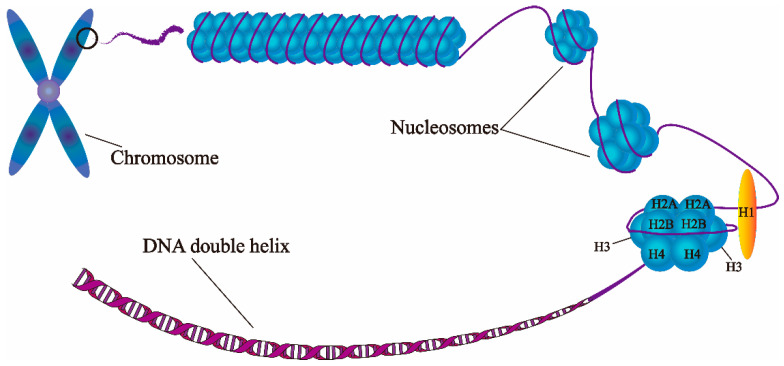
A model of chromatin.

Plants respond and adapt to drought stress through a series of transcriptional activations or repressions [24,30]. These transcriptional activations or repressions rely on the complex chromatin structure changes caused by epigenetic modifications [25,26,31,32]. Epigenetic mechanisms regulate or modify gene expression without changes in the underlying DNA sequence. These mechanisms include DNA methylation, histone modifications, histone variants, and chromatin remodeling, which modify chromatin structure and affect gene transcription [25,26]. Chromatin is a highly ordered structure composed of DNA, histones and other chromosomal proteins. The basic unit of chromatin is the nucleosome, which consists of 147 bp of DNA wrapped 1.7 times around an octamer protein core of histones containing two copies of H2A, H2B, H3 and H4 (Figure 2) [25,33]. There are 14 contact points between histones and DNA, contributing to the compactness and stability of chromatin [34]. The N-terminal tails of the histone proteins protrude from the nucleosome core and are subjected to post-translational modifications (PTMs), such as acetylation, methylation, and phosphorylation [35]. There are at least eight types of histone N-tail modifications, termed “epigenetic marks” or “histone code” [26]. Histone tails have also been considered as sites of integration of signals transduced from transacting stimuli, such as stresses. Signaling events may act directly on histone N-terminal tails to modify chromatin, such as acetylation of histone lysines, and thus regulate chromatin structure and gene expression [36].

**Figure 3:**
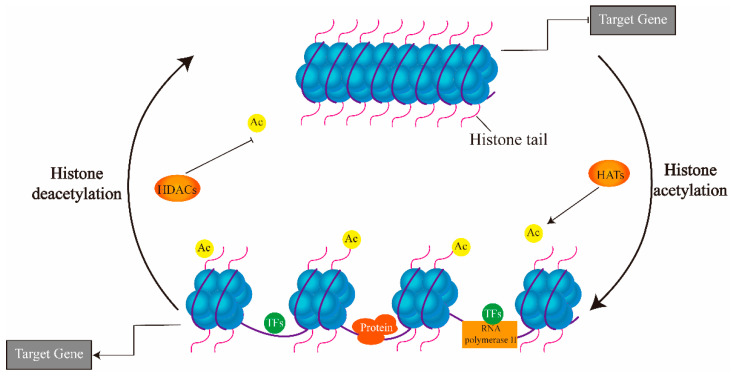
Regulation of histone acetylation by HATs and HDACs.

Acetylation and methylation at histone lysine residues are two of the most studied marks [25,26,37]. The levels of acetylated histones are dynamic and depend on the combined action processes catalyzed by two groups of enzymes, histone acetyltransferases (HATs) and histone deacetylases (HDACs) (Figure 3) [37]. HATs catalyze the transfer of the acetyl group from acetyl coenzyme-A onto the ε-amino acid group of lysine residues, making histones less positively charged and therefore less attracted to the negatively charged DNA resulting in an “open” chromatin [33,38,39]. HDACs do the opposite—removing the acetyl group from the lysine residues to “close” chromatin [33,40]. At any given time and place, the quantity of the acetylated histones, quantifiable by ChIP (Chromatin immunoprecipitation), represents the net action of histone acetylation [13]. The quantity of the net acetylation determines whether the chromatin is open or closed, thereby controlling access of DNA-binding proteins, such as transcription factors, for transcriptional activation [13].

Modification-specific antibody-based ChIP coupled with sequencing (ChIP-seq) is highly reliable for the genome-wide analysis of histone modification patterns and has been used for plants, which include Arabidopsis, rice, maize, and *Populus trichocarpa* [13,41,42,43]. These studies revealed some common features regarding genome-wide modification patterns. Acetylation is enriched at sites in the promoter and at the 5’ end surrounding the transcription start site and that promoter regions of active genes have reduced nucleosome occupancy.

## 4. HATs and HDACs in Plants

The level of histone acetylation is determined by the regulation of histone acetyltransferases (HATs) and histone deacetylases (HDACs), and the antagonism between HATs and HDACs leads to dynamic control of chromatin structure [39,44]. HATs are responsible for adding acetyl groups to histones causing increasing histone acetylation; this process results in gene activation [33,38]. HDACs are responsible for removing acetyl groups added by HATs to reduce histone acetylation, and HDACs always associate with gene repression [33,40,45]. Evidence in previous research indicates that HATs and HDACs always target the lysine residues of H3 and H4, such as H3K9, H3K14, H3K18, H3K23, H3K27, H3K36, H4K5, H4K8, H4K12 and H4K16 to maintain the dynamic balance of histone acetylation in plants [33]. HATs have been identified in many plants (Table 1). According to their sequence characterization, HATs have been divided into four distinct families, such as GNAT (GNAT: GCN5-related N-terminal acetyltransferases), MYST (MYST: MOZ-YBF2/SAS3-SAS2/TIP60), CPB (p300/CREB binding protein) and TAFII250 (TATA-binding protein associated factor) [46]. HDACs have also been identified in plants (Table 1) and have been divided into three sub-families, i.e., Reduced Potassium Dependency 3/histone deacetylase 1 (RPD3/HDA1), Silent Information Regulator 2 (SIR2) and the plant-specific histone deacetylase 2 (HD2) [46]. Different families of HATs and HDACs have distinct protein domains that are responsible for regulating levels of acetylation and, thus, gene expression in many biological processes, including drought response and resistance [46].

## 5. Histone Acetylation Level in Drought Responses

Histone acetylation always acts as an active regulator cooperating with other factors, such as transcription factors and other proteins [67,68]. The levels of histone acetylation are dynamic and associate with the combined action processes catalyzed by HATs and HDACs [39]. Drought stress often induces histone acetylation changes in “drought-responsive” genes and other genes, causing genome-wide histone acetylation alterations in plants.

### 5.1. HATs

HATs are responsible for increasing histone acetylation and associate with gene activation. Recent evidence shows that drought stress induces different expression patterns of HAT genes, resulting in changes in the levels of histone acetylation of drought response genes. In rice, the expression of *OsHAG702*, *OsHAG703* and *OsHAM701* was significantly increased after drought treatment [59]. In Chinese cabbage, the expression of *BraHAC1*, *BraHAC2*, *BraHAC3*, *BraHAC4*, *BraHAC7*, *BraHAG2*, *BraHAG5*, *BraHAG7* and *BraHAF1* was increased after drought treatment [48]. In *Brachypodium distachyon*, the expression of five *BdHAT* genes, i.e., *BdHAG1*, *BdHAG3*, *BdHAC1*, *BdHAC4* and *BdHAF1* was increased after drought treatment [47].

Up- or down-expression of HAT genes can easily lead to different expression of drought response genes and variations of drought-resistant characteristics in plants. *TaHAG2*, *TaHAG3* and *TaHAC2* of HAT genes were up-regulated under drought stress in a higher drought resistance wheat variety called BN207, but not in other drought-sensitive varieties. This result suggests that the differential expression of these HAT genes might be responsible for the higher drought resistance of BN207 [64]. In *P. trichocarpa*, RNA interference-mediated downregulation of the HAT gene PtrGCN5-1 resulted in reduced expression of drought response genes and highly drought-sensitive plants [13].

The above studies describe types of expression alterations of HAT genes in plants under drought stress. A recent work uncovered a detailed mechanism of a HAT complex which is recruited by a transcription factor to enhance enrichment of H3K9ac and RNA polymerase II specifically at related genes for drought response [13]. H3K9ac, a general chromatin marker of gene activation, is one of the most extensively studied histone acetylation marks in vascular plants [13,67,69,70]. Hyperacetylation of H3K9 is almost associated with the activation of transcription, whereas hypoacetylated histones are accompanied by transcriptional repression [13,70,71,72]. The HAT family gene GCN5 is a well-known enzymatic protein responsible for the lysine acetylation of histone H3 and H4 [73]. In *P. trichocarpa*, drought stress results in histone acetylation changes at the whole genome level. Consistent with H3K9ac being a gene activation mark, most up-regulated genes induced by 5 days (D5) or 7 days (D7) of drought treatment exerted increased H3K9ac level at their promoter regions, and decreased H3K9ac level at promoters of down-regulated genes. The authors identified nearly 4000 (3994, D5; 3498, D7) up-regulated genes with increased H3K9ac level and down-regulated genes with decreased H3K9ac level. Motif analysis of these differentially expressed genes (DEGs) revealed that the ABRE motif for the ABA-Responsive Element Binding protein (AREB1) was most significantly enriched within the H3K9ac-associated promoters [14,23]. A series of molecular biology techniques demonstrated that the AREB1 transcription factor interacts with the GCN5-ADA2b histone acetyltransferase complex and recruits the complex to drought-responsive genes, such as PtrNAC006, 007 and 120 genes, through binding to ABRE motifs, resulting in enhanced H3K9ac and RNA polymerase II enrichment for activating expression of the *PtrNAC006*, *007* and *120* genes, thereby allowing *P. trichocarpa* for adaptation to drought stress. This research suggests that transcription factors interplay and coordinate with H3K9ac in response to drought stress, demonstrating a link between transcriptional and epigenetic regulations in controlling drought response and resilience [13].

### 5.2. HDACs

Studies have reported that drought stress induces different expression patterns of *HDAC* genes to influence levels of histone acetylation at drought-related genes and change the drought resistance of plants. Yang et al. revealed that the expression levels of nine examined *GmHDACs* (*GmHDA6*, *GmHDA8*, *GmHDA13*, *GmHDA14*, *GmHDA16*, *GmSRT2*, *GmSRT4*, *GmHDT2*, *GmHDT4*) were decreased by drought treatment in soybean [60]. In rice, the transcriptional levels of two *HDAC* genes (*OsHDA703*, *OsHDA710*) were significantly decreased, while *OsHDA704*, *OsHDA705*, *OsHDA706*, *OsHDA711*, *OsHDA712*, *OsHDA713*, *OsHDT701* and *OsSRT702* were significantly increased [59]. In *Dendrobium officinale*, *HDAC* genes displayed different expression patterns in different tissues. *HDAC* genes also displayed different transcriptional level to respond to drought. For example, *DoHDA10* and *DoHDT4* were highly up-regulated after PEG-mediated drought treatment, while other *HDAC* genes were not changed [52]. Wei et al. studied the roles of six *HDACs* in response to drought stress in kenaf and found up-regulation of all these *HDACs* after PEG treatments [74]. In addition, high acetylation levels of H3K and low acetylation levels of H3K27 and H4K5 were observed following a lower PEG treatment. Meanwhile, acetylation levels of H3K9, H3K27 and H4K5 were all reduced in response to a higher PEG treatment [74].

As drought stress induces different expression patterns of *HDACs*, disturbed expression of *HDACs* always leads to drought-resistant characteristics changes. A tomato *HDAC* gene, *SlHDA5*, a gene expressed ubiquitously in different tissues and development stages, is induced by ABA. *SlHDA5*-RNAi transgenic plants were more sensitive to drought stress than wild type plants [75]. In Arabidopsis, mutants of *HDA19* were more drought-resistant than wild type plants [76]. Additionally, overexpression plants of another *HDAC* gene, *AtHD2D*, exhibited higher degrees of tolerance to abiotic stresses, including drought [77]. In addition, a drought-responsive gene in 84 K poplar (*Populus alba* × *Populus glandulosa*), named *84KHDA903*, was transformed into tobacco for understanding its function in stress responses. The *84KHDA903*-expressing tobacco were more drought-tolerant and showed strong capacity to recover after drought stress [78]. In *Brachypodium distachyon*, overexpression plants of a *HDAC* gene *BdHD1* displayed a hypersensitive phenotype to ABA and exhibited better survival under drought conditions. Consistently, the *BdHD1*-RNAi plants were insensitive to ABA with low survival under drought treatment [79]. ChIP-seq analysis showed that overexpression of *BdHD1* led to lower H3K9 acetylation at the transcriptional start sites of 230 drought-specific genes, and expression of these genes was repressed. The study concluded that BdHD1 changes the level of histone acetylation at drought-responsive genes to influence drought resistance of *Brachypodium distachyon* [79].

**Figure 4:**
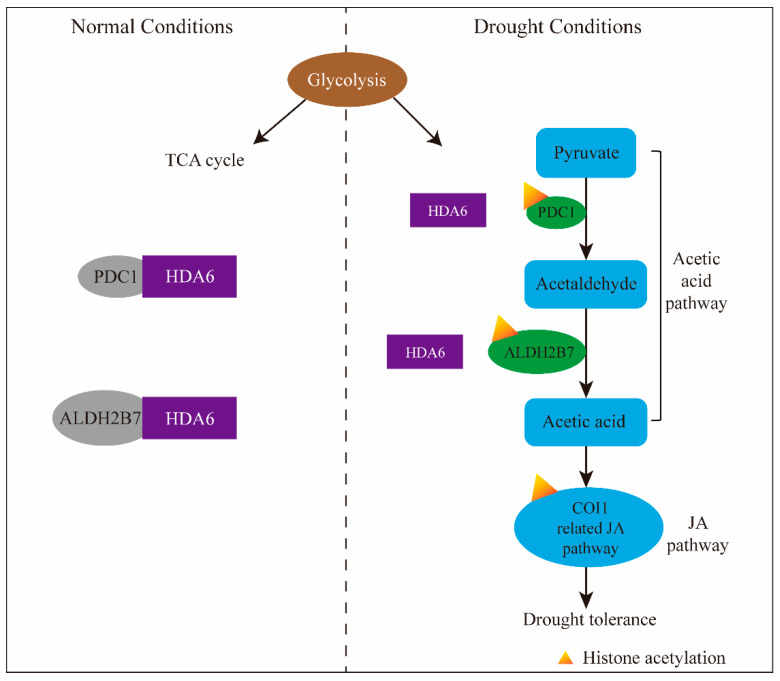
A model of HDA6-mediated glycolysis-acetic acid-JA drought response signaling pathway.

In addition to the work described above, some detailed mechanisms of HDACs responding to drought stress have been revealed, for HDA6, HDA9 and HDA15. In Arabidopsis, HDA6 works as an ON/OFF switch of a complex drought-responsive pathway [19]. HDA6 triggers a dynamic metabolic flux conversion from glycolysis to acetate synthesis to stimulate the jasmonate (JA) signaling pathway to develop drought tolerance (Figure 4) [19]. In plants, the acetic acid biosynthesis pathway is considered the conversion from pyruvate to acetic acid. Pyruvate decarboxylase PDC1 initiates the first step to redirect the metabolic flux from pyruvate in glycolysis to acetaldehyde. Additionally, acetaldehyade dehydrogenase ALDH2B7 converts acetaldehyde to acetate subsequently [80]. Under normal conditions, HDA6 binds to *PDC1* and *ALDH2B7* to repress the expression of them by reducing histone H4 acetylation (H4Ac) enrichment at their transcribed gene body regions. Under drought conditions, HDA6 dissociates from *PDC1* and *ALDH2B7* to trigger increased H4Ac enrichment and transcriptional upregulation of these two genes. As a result, the acetic acid biosynthesis pathway is initiated, pyruvate in glycolysis is converted to acetaldehyde by PDC1, and acetaldehyde is converted to acetate by ALDH2B7. The accumulation of acetate is essential for drought tolerance improvement. Evidence suggests that the drought tolerance of Arabidopsis pretreated with exogenous acetic acid exhibited striking improvement [19]. In the study, 357 genes were induced under drought, specifically in the acetic acid-pretreated plants, and JA response genes were enriched among these genes [19]. In acetic acid-treated plants, both the expression of the JA biosynthetic enzyme AOC3 and the biosynthesis of JA and jasmonoyl–isoleucine (JA-Ile) were greatly induced [19]. This means that HDA6 triggers the acetic acid biosynthesis, accumulation of acetic acid stimulates the JA signaling pathway initiation to enhance plant drought tolerance. In this process, the JA receptor COI1 is essential. The interaction between HDA6 and COI1 suggests that HDA6 regulates JA signaling pathways cooperatively with COI1. Additionally, the chromatin status of COI1 target genes might be influenced by histone acetylation [19].

The Arabidopsis HDA9, a RPD3-type histone deacetylase, is involved in modulating plant responses to drought stress by regulating histone acetylation levels of many stress-responsive genes [81]. The loss-of-function mutants of *HDA9* were sensitive to drought stress, but insensitive to ABA [82,83]. HDA9 can work together with Powerdress (PWR) protein to induce expression of ABA-responsive genes, including RD29A, RD29B, and COR15A for plant ABA response and drought tolerance [82]. On the other hand, the ABA INSENSITIVE 4 (ABI4) transcription factor, associated with regulation of plant sensitivity to ABA, interacts with PWR and HDA9 to influence the accumulation of ABA and plant drought response [82,83]. CYP707A1 is key enzyme which is responsible for converting active ABA to inactive ABA [83]. Under normal conditions, *CYP707A1* expresses to keep a low level of ABA in plants. Under drought conditions, PWR-HDA9-ABI4 complex binds to the promoter of *CYP707A1* and reduces the histone acetylation levels for transcriptional repression, which results in accumulation of ABA to initiate drought response [82,83].

Based on another study, HDAC gene HDA15 is recruited by a transcription factor to influence ABA signaling response in Arabidopsis [84]. MYB96, an ABA-inducible transcription factor, can bind to the promoters of many ABA-responsive genes for expression regulation to optimize stress tolerance [85,86,87]. In the presence of moderate ABA, MYB96 works as a transcriptional activator to promote expression of ABA-induced genes, such as RD29A. However, under high concentrations of ABA, MYB96 interacts with HDA15 and presents as a transcriptional repressor. In the presence of high ABA, MYB96 specifically binds to promoters of RHO GTPASE OF PLANTS (ROP) genes, negative regulators of ABA responses, and recruits the HDA15 protein to remove acetyl groups of histone H3 and H4 from the cognate regions. For this reason, the expression of ROP genes is repressed and the ABA signaling response is enhanced [84].

## 6. Conclusions

Drought stress often induces histone acetylation changes for many drought-responsive genes to trigger transcriptional activations or repressions for drought response/resistance in plants. The changes in histone acetylation are dynamic and determined by HATs and HDACs. HATs are responsible for increasing histone acetylation at related genes to activate transcription. HDACs are responsible for reducing histone acetylation to repress transcription. Both HATs and HDACs have been identified in many plants. HATs and HDACs work in many drought response signal pathways, including ABA, acetic acid and JA pathways, by changing the level of histone acetylation at targeted pathway genes. Other regulatory factors, such as transcription factors and protein, work cooperatively with HATs and HDACs to influence the levels of histone acetylation. While histone acetylation affecting the expression of many genes has been revealed, knowledge of detailed molecular and genetic mechanisms underlying such affects is still lacking. Crosstalk between histone acetylation and other epigenetic marks is worthy of being explored.

## Figures and Tables

**Table 1:** Identification of HATs and HDACs in different plants.

Plant	HATs		HDACs	
	Number	Reference	Number	Reference
*Arabidopsis thaliana*	12	[46]	18	[46]
*Brachypodium distachyon*	8	[47]	-	-
Chinese Cabbage (*Brassica rapa*)	15	[48]	20	[49]
Cotton (*Gossypium raimondi* and *Gossypium arboretum*)	9	[50]	-	-
Cotton (*Gossypium hirsutum*)	18	[50]	30	[51]
*Dendrobium officinale*	-	-	14	[52]
Grape (*Vitis vinifera*)	7	[53]	13	[53]
Litchi (*Litchi chinensis Sonn. cv. Feizixiao*)	6	[54]	11	[54]
Maize (*Zea mays*)	-	-	18	[55]
*Marchantia polymorpha*	7	[56]	12	[56]
Rice (*Oryza sativa*)	8	[57]	18	[58]
7 Gramineae genomes (*Oryza sativa*)	37	[59]	110	[59]
Soybean (*Glycine max*)	-	-	28	[60]
Sweet orange (*Citrus sinensis*)	50	[61]	16	[61]
Tea (*Camellia sinensis* L. *O. Kuntze*)	-	-	18	[62]
Tomato (*Solanum lycopersicum*)	32	[63]	15	[63]
Wheat (*Triticum aestivum*)	30	[64]	53	[64]
Wheat (*Triticum aestivum*)	31	[65]	49	[66]
